# Molecular characterization of recombinant LSDV isolates from 2022 outbreak in Indonesia through phylogenetic networks and whole-genome SNP-based analysis

**DOI:** 10.1186/s12864-024-10169-6

**Published:** 2024-03-04

**Authors:** Indrawati Sendow, Irene Kasindi Meki, Ni Luh Putu Indi Dharmayanti, Heri Hoerudin, Atik Ratnawati, Tirumala Bharani K. Settypalli, Hatem Ouled Ahmed, Harimurti Nuradji, Muharam Saepulloh, Rahmat Setya Adji, Nuha Fairusya, Faralinda Sari, Katamtama Anindita, Giovanni Cattoli, Charles Euloge Lamien

**Affiliations:** 1 Research Center for Veterinary Science, Research Organization for Health, National Research and Innovation Agency, West Java, Indonesia; 2 Animal Production and Health Laboratory, Joint FAO/IAEA Centre of Nuclear Techniques in Food and Agriculture, Department of Nuclear Sciences and Applications, International Atomic Energy Agency, Wagramer Strasse 5, A-1400 Vienna, P.O. Box 100, Austria; 3 Regional Livestock Services, Riau Province, Indonesia; 4 Disease Investigator Center Bukit Tinggi, West Sumatera, Indonesia

**Keywords:** LSDV, Whole genome sequencing, Recombinant, Phylogenetic network, SNP

## Abstract

Lumpy skin disease (LSD) is a transboundary viral disease of cattle and water buffaloes caused by the LSD virus, leading to high morbidity, low mortality, and a significant economic impact. Initially endemic to Africa only, LSD has spread to the Middle East, Europe, and Asia in the past decade. The most effective control strategy for LSD is the vaccination of cattle with live-attenuated LSDV vaccines. Consequently, the emergence of two groups of LSDV strains in Asian countries, one closely related to the ancient Kenyan LSDV isolates and the second made of recombinant viruses with a backbone of Neethling-vaccine and field isolates, emphasized the need for constant molecular surveillance. This current study investigated the first outbreak of LSD in Indonesia in 2022. Molecular characterization of the isolate circulating in the country based on selected LSDV-marker genes: RPO30, GPCR, EEV glycoprotein gene, and B22R, as well as whole genome analysis using several analytical tools, indicated the Indonesia LSDV isolate as a recombinant of LSDV_Neethling_vaccine_LW_1959 and LSDV_NI-2490. The analysis clustered the Indonesia_LSDV with the previously reported LSDV recombinants circulating in East and Southeast Asia, but different from the recombinant viruses in Russia and the field isolates in South-Asian countries. Additionally, this study has demonstrated alternative accurate ways of LSDV whole genome analysis and clustering of isolates, including the recombinants, instead of whole-genome phylogenetic tree analysis. These data will strengthen our understanding of the pathogens’ origin, the extent of their spread, and determination of suitable control measures required.

**Supplementary Information:**

The online version contains supplementary material available at 10.1186/s12864-024-10169-6.

## Introduction

Lumpy skin disease virus (LSDV) is a member of the *Capripoxvirus* (CaPV) genus that has a linear double-stranded DNA genome [1]. The virus causes lumpy skin disease (LSD) in cattle and water buffaloes [2]. The CaPV genus comprises two other species, the sheeppox virus (SPPV) and goatpox virus (GTPV), which are antigenically and genetically closely related to LSDV [1, 3]. LSD infections are associated with high morbidity (up to 100%) and low mortality (< 10%), with a significant economic impact due to losses related to animal production, restrictions to trade, and treatment/vaccination costs, and therefore a significant World Organisation for Animal Health (WOAH) notifiable disease [2, 4]. Clinically sick animals present symptoms such as fever, enlarged lymph nodes, nasal discharge, and nodular skin lesions [5, 6].

LSDV is transmitted by blood-feeding vectors such as stable flies, mosquitoes, and ticks [7–9]. This may have facilitated its spread from the endemic region in Africa to the Middle East, Europe, and South Asia in the past ten years [10]. Other modes of transmission, such as direct contact, seminal, and intrauterine transmission, have also been demonstrated experimentally [11–13]. LSD control measures include cattle movement/trade restriction, vector control, disinfection, or culling of the animals showing clinical signs [2, 14], with the most effective control strategy being vaccination using live attenuated vaccines, such as those derived from the Neethling strain [15–17].

Understanding the molecular and genetic epidemiology of CaPVs is a prerequisite for better management and control of LSD, given the antigenic similarity of CaPVs that challenges their distinction by serological tests. Among several molecular-based techniques for genetically distinguishing CaPVs include, the sequence analysis of genes encoding the RNA polymerase 30 kDa subunit (RPO30), the G-protein-coupled receptor (GPCR), the CaPV homolog of the variola virus B22R, and the EEV glycoprotein that possess specific signatures that can differentiate the CaPVs [18–21]. These genes could also help distinguish live attenuated vaccines from field isolates and the recombinant LSDV strains. However, a whole genome-based approach is preferable as it enables proper recombination analysis which has been done for the recently reported recombinants [22, 23]. Nevertheless, phylogenetic tree construction using recombinants’ whole genome is not suitable/accurate since the analysis process considers mainly the mutations and speciation events and does not account for recombination between the sequences or ancestor sequences [24]. Therefore, alternative tools such as restricting the analysis to parts of the virus genome, or phylogenetic networks and single nucleotide polymorphism (SNP) genotyping, which consider events like recombination/hybridization have been shown as more appropriate for whole genome analysis of recombinants [25–27].

LSD was first reported in Asia in 2019 in India, Bangladesh, and China and subsequently spread to other Asian countries such as Nepal, Myanmar, Thailand, and Vietnam [28]. Phylogenetic analysis of the Asian LSDV isolates has demonstrated that the isolate circulating in South-Asian countries resembles the ancient LSDV isolate from Kenya [29–31], while the isolates in Southeast and Eastern Asia are recombinant viruses with Neethling vaccine strain and field isolates backbones [23, 32–35]. In Indonesia, the initial suspected clinical cases of LSD were reported in November 2020 cattle and buffaloes from Kampar and buffaloes from Kuansing districts, Riau Province. In 2021, more suspicions were reported in cattle from Padang Pariaman district in North Sumatera (January) and in a buffalo from Muara Bungo district (August). However, none of the suspected LSD cases from 2020 to 2021 could be confirmed by either serological (Capripox ELISA) or molecular tests (Polymerase chain reaction (PCR). In February 2022, the disease spread to cattle from Indragiri Hulu district, where samples were collected and confirmed by PCR at the Research Centre for Veterinary Science (RCVS), National Research and Innovation Agency, enabling the notification to WOAH on February 2022 [36]. This study aims to provide clinical evidence of the first outbreak of LSD in Indonesia in 2022 and the molecular characterization of the LSDV isolate circulating in the country based on selected LSDV-marker genes and whole genome analysis. Due to the recent emergence of LSDV recombinants that make it challenging (less accurate) to cluster LSDV isolates using whole genome phylogenetic trees, the study will also explore some alternative approaches based on existing tools for better accuracy in LSDV whole genome-based clustering.

## Results

### LSD outbreak investigation and clinical signs

**Fig. 1:**
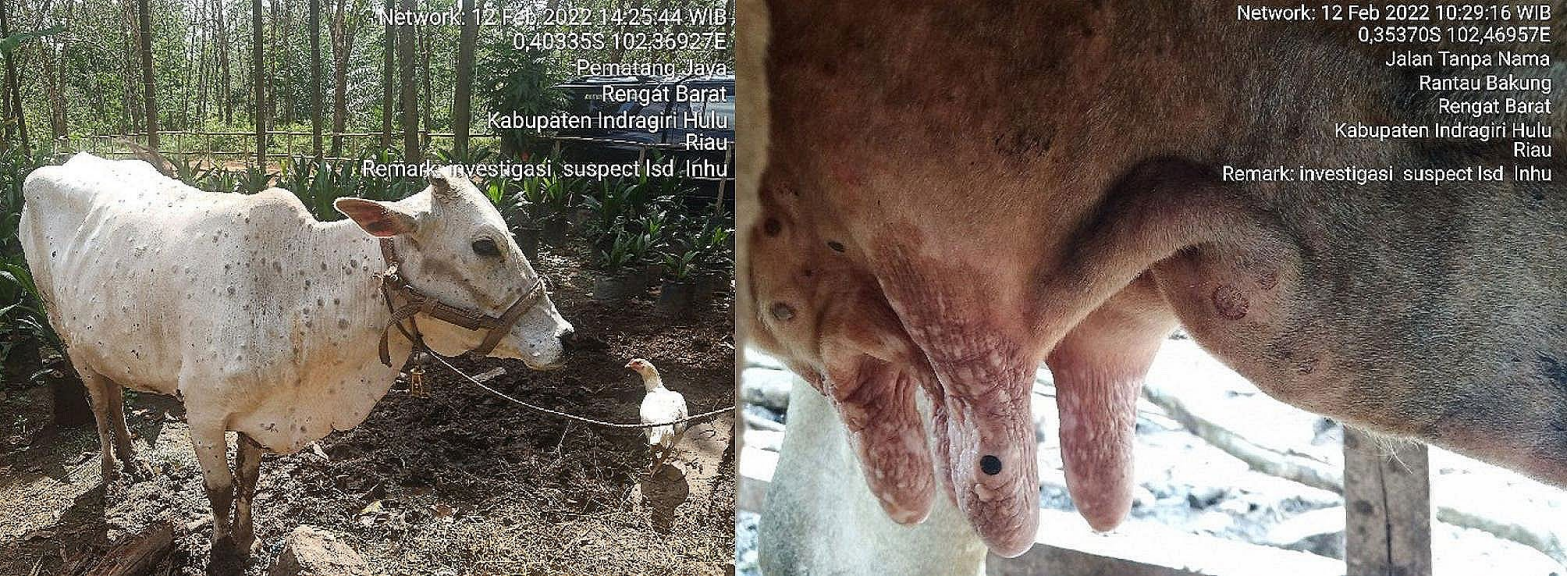
Clinical signs of LSD suspected cases in Indonesia; nodules on cattle from Indragiri Hulu district

The suspected LSD infected animals presented nodular lesions on different body parts, nasal discharge, and fever (Fig. 1). The morbidity and mortality rates recorded in the Indragiri Hulu district by February 2022 were 28.04% (30/107) and 1.87% (2/107), respectively.

### Molecular detection and diagnosis of LSDV

CaPV genome was detected in ten (83.33%) out of the twelve samples collected from 2022 suspected cattle cases in Indragiri Hulu district, Indonesia, by RT-PCR using the Bowden et al. protocol [6]. Further analysis of the positive samples, using HRM assay, confirmed that the suspected cases were infected with LSDV (Additional file 1: Table s1).

### Targeted gene sequence and phylogenetic analysis

For five samples, the targeted fragments were successfully amplified and sequenced, and the assembled contigs of 606, 1146, 327, and 832 bp for the RPO30, GPCR, EEV, and B22R genes, respectively, were deposited in the GenBank with accession numbers OR246967 to OR246986.

**Fig. 2:**
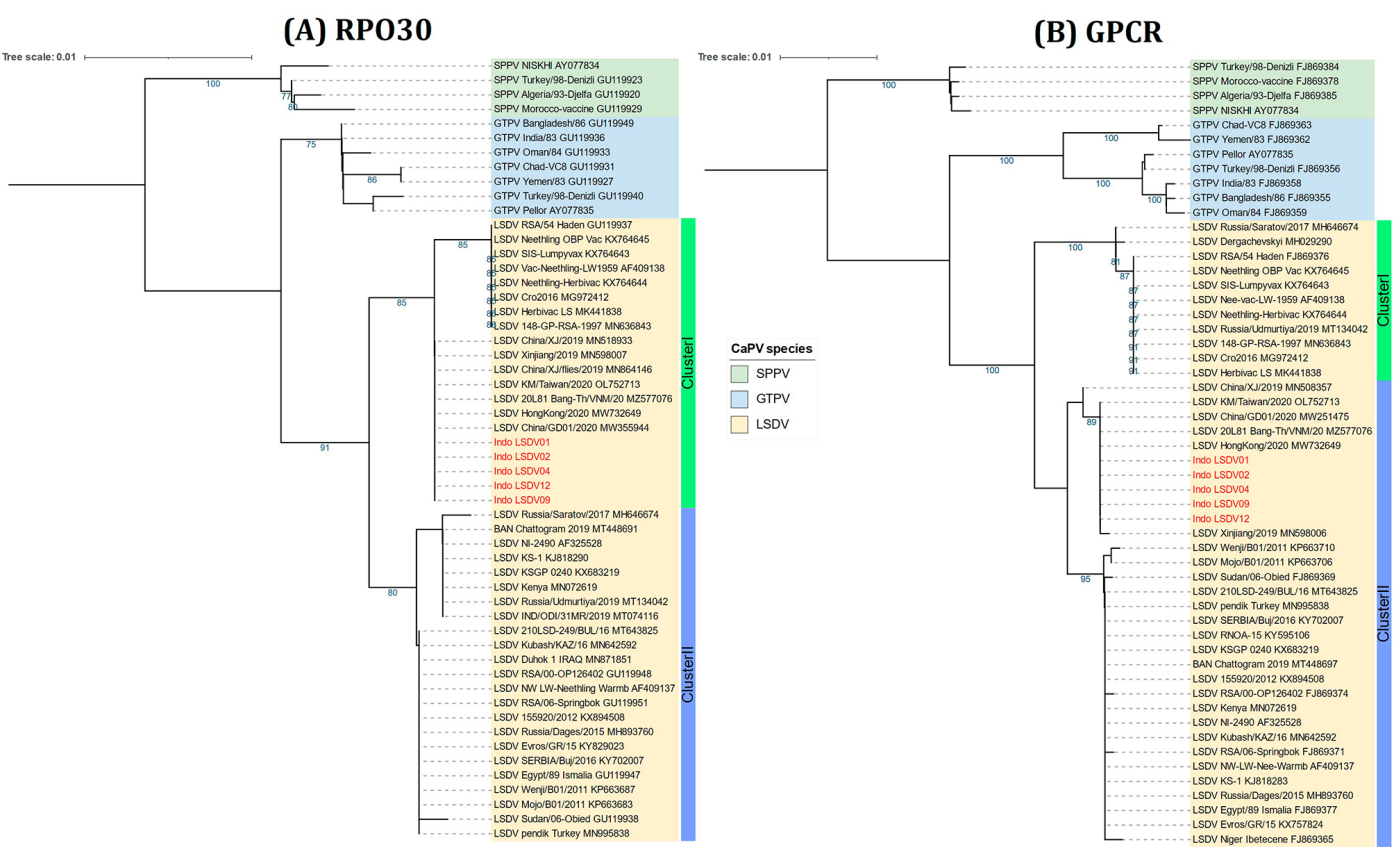
Neighbour-joining tree based on the complete (**A**) RPO30 and (**B**) GPCR gene sequences of CaPVs with LSDVs from Indonesia (in red) visualized on iTOL. The evolutionary distances were computed using the Maximum Composite Likelihood method with 1000 bootstrap replicates on MEGA X

The multiple sequence alignments showed that all five Indonesia LSDV samples were 100% identical based on RPO30, GPCR, EEV, and B22R gene sequences. Phylogenetic analysis of the complete RPO30 gene sequences showed that Indonesia sequences were grouped in LSDV Cluster I together with LSDV Neethling-derived vaccines and other LSDV isolates from East and Southeast Asia, such as China, Taiwan, Vietnam, and Thailand (Fig. 2A). However, the GPCR gene sequences grouped the Indonesia sequences in LSDV Cluster II with the common LSDV field isolates, the historical LSDVs from Kenya, and LSDV KSGP-0240 derived vaccine, yet with the other East and Southeast Asia isolates (Fig. 2B).

**Fig. 3:**
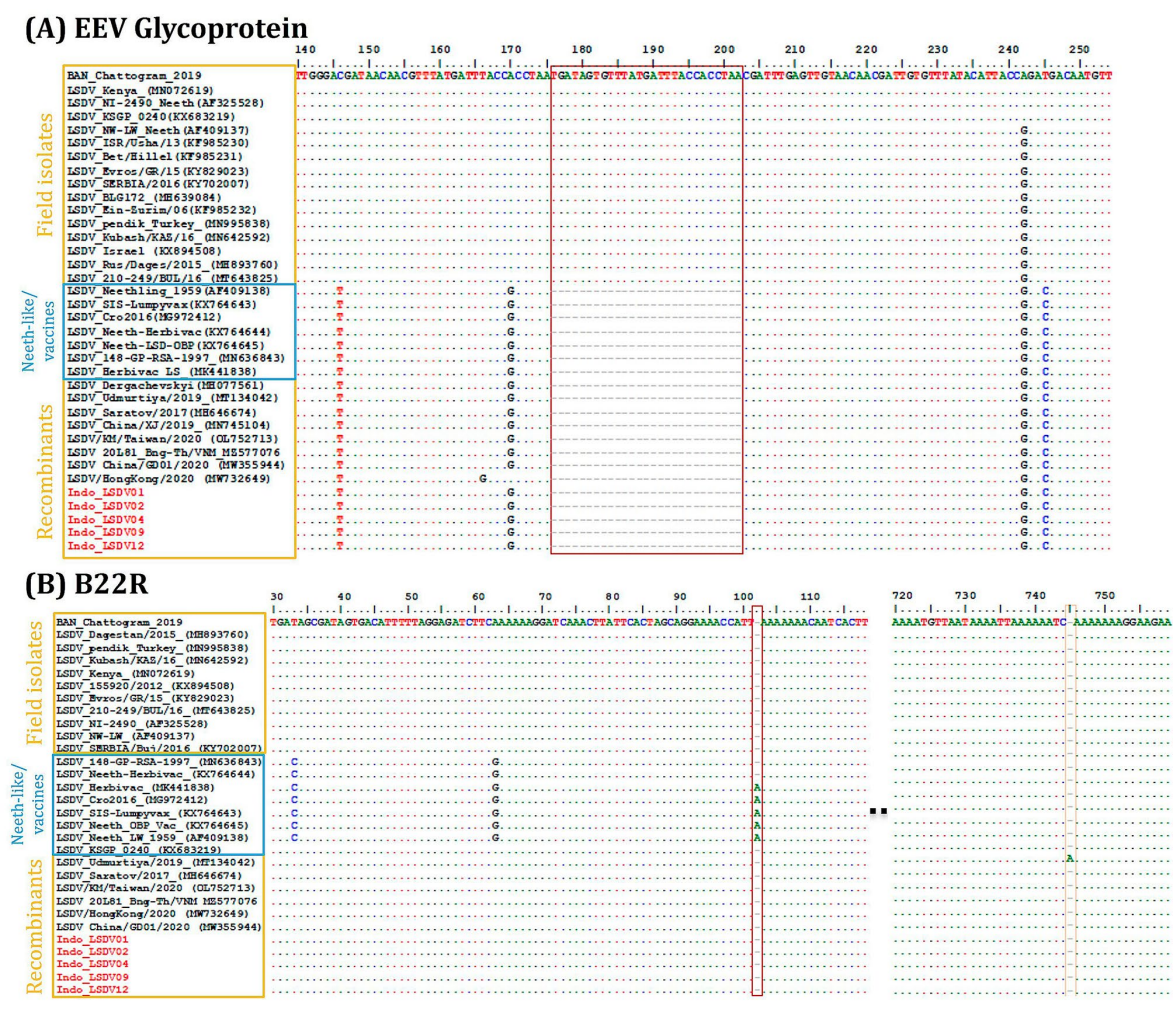
Multiple sequence alignment of (**A**) the partial sequences of the EEV glycoprotein gene showing the 27-nucleotide deletion highlighted in the block found in Indonesia isolates and (**B**) partial sequences of the B22R gene showing the nucleotide insertion (in blocks) in LSDV_Neethling and LSDV_KSGPO-240 vaccines that are absent in Indonesia isolates (in red). The dots indicate the identical nucleotides in the alignment

The multiple sequence alignment of the partial EEV glycoprotein gene showed that Indonesian LSDV isolates are identical in this region of the genome to LSDV isolates from China, Vietnam, Taiwan, and LSDV Neethling-derived vaccines that have a 27-nucleotide deletion (175–201) (Fig. 3A). The B22R sequence alignment showed that part of the Indonesian sequence was identical to the historical LSDVs from Kenya (353 bp) and the other to the LSDV Neethling-derived vaccine (479 bp). However, the B22R of the Indonesian LSDVs differed from LSDV Neethling and LSDV KSGP-0240 derived vaccines due to one nucleotide InDel at positions 102 and 745, respectively (Fig. 3B).

### LSDV whole genome SNPs and recombination analysis

The assembled LSDV genomes were 151,010 bp long for each of the two Indonesian isolates; LSDV_Indonesia_2022_S1 and LSDV_Indonesia_2022_S4, with a mean coverage of 2,856 ± 814 and 637 ± 174 respectively. These genomes comprised 158 predicted ORFs each (Additional file 1: Table s2). Sixty-eight (68) of the predicted ORFs were 100% homologous to both LSDV_NI-2490 (LSDV Cluster II) and LSDV_Neethling_vaccine_LW_1959 (LSDV Cluster I) reference strains, while 26 ORFs were only 100% identical to LSDV_NI-2490 and 55 ORFs to LSDV_Neethling_vaccine_LW_1959. The remaining 9 ORFs were 99.5-99.9% homologous to LSDV_NI-2490 and LSDV_Neethling_vaccine_LW_1959 reference strains (Additional file 1: Table s3). NCBI nucleotide blast search based on the whole genome revealed that over the aligned genome fragment, the Indonesia LSDV genome shared 100% nucleotide identity to LSDVs from Thailand (OM033705.1), Vietnam (MZ577076.1) and China (OM803092.1), and 99.98% and 99.94% to LSDVs from Taiwan (OL752713.2) and HongKong (MW732649.1) respectively. In addition, the Indonesian LSDVs shared 99.56% and 99.25% nucleotide identity to LSDV_Neethling_vaccine_LW_1959 and LSDV_NI-2490, respectively. The shared identities between the Indonesian LSDV genomes and the recombinant LSDVs from Russia were 99.41% to Saratov_2017 (MH646674_1), 99.51% to Udmurtiya_2019 (MT134042_1), 99.54% to Saratov_2019 (OM530217_1) and 99.64% to Tyumen_2019 (OL542833_1).

**Fig. 4:**
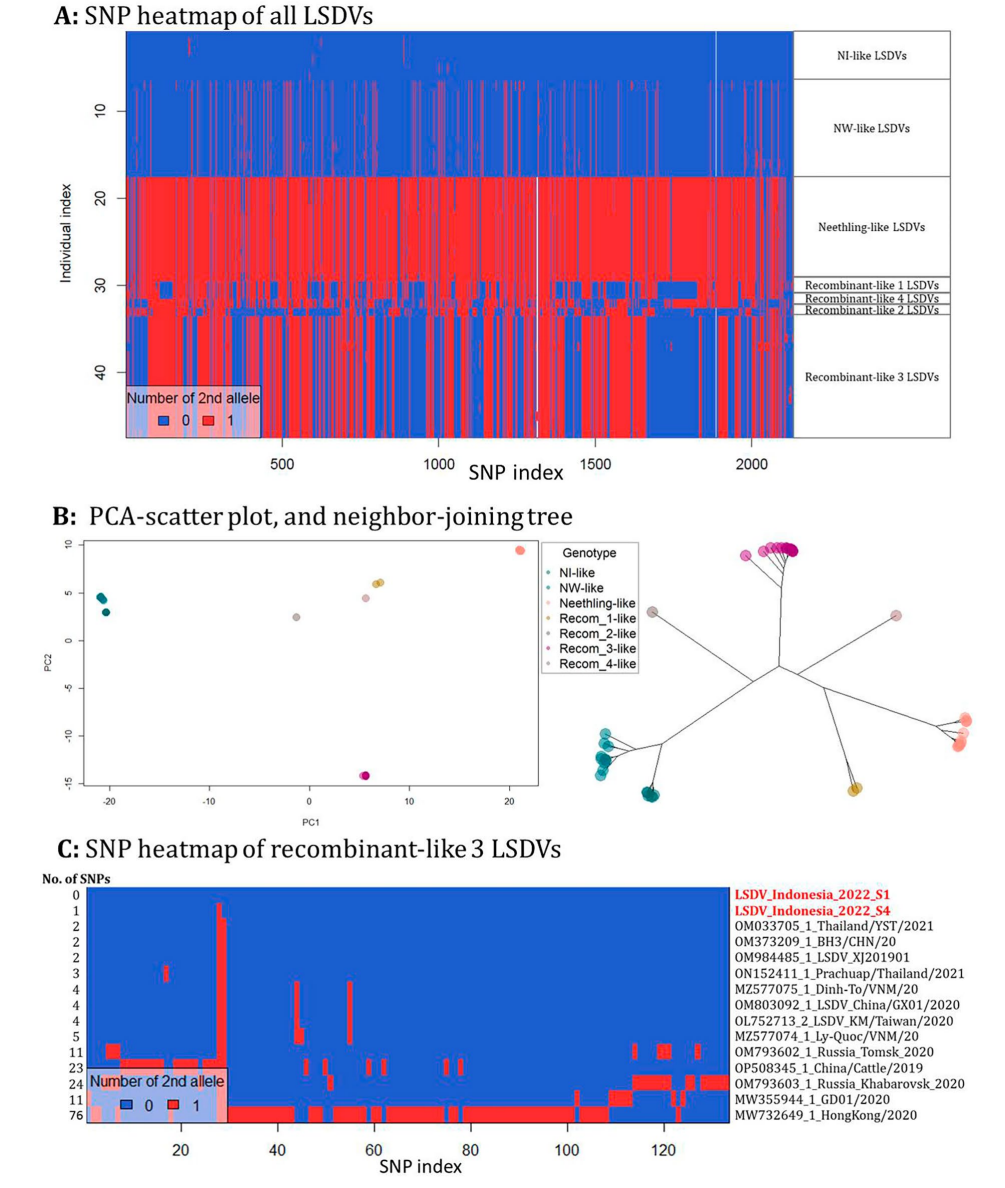
Analysis of SNPs extracted from LSDV genome alignments. (**A**) SNP distribution heatmap, (**B**) PCA-scatter plot, and neighbor-joining tree based on the PCA scores of the SNP data of all LSDV complete genomes, (**C**) SNP distribution heatmap of recombinant_3-like LSDV genomes

SNP analysis of the aligned LSDV genomes relative to the LSDV_NI-2490 (AF325528.1) reference genome revealed 2145 SNPs distributed across the genome. The heatmap based on the SNP index showed seven clusters of LSDV genotypes; NI-like (related to the ancient NI-2490 LSDV), NW-like (commonly circulating LSDV field isolates), Neethling-like (related to Neethling_vaccine) and four recombinant clusters, recombinant_1-like (Saratov_Russia_isolates), recombinant_2-like (Udmurtiya_Russia_isolate), recombinant_3-like (East and Southeast Asia isolates) and recombinant_4-like (Tyumen_Russia_isolate) (Fig. 4A). The Indonesian LSDVs clustered with the recombinant_3 like LSDVs, together with LSDV isolates from Thailand, Vietnam, Taiwan, and China. The seven clusters of LSDVs based on the SNPs profile were also differentiable by the PCA scatterplot and the neighbor-joining tree (Fig. 4B). Further SNP analysis of the recombinant_3-like LSDVs relative to the LSDV_Indonesia_2022_S1, detected 133 SNPs across the 15 aligned genomes. While there was one SNP between the two Indonesian isolates, the heatmap revealed 2–5 SNPs between the Indonesia LSDVs and other East and Southeast Asian isolates such as Thailand_OM033705_1, China_OM373209_1/OM984485_1, Vietnam_MZ577075_1, and Taiwan_OL752713_2. The highest number of SNPs were detected in Isolates from China, MW355944_1_LSDV/GD01/2020 (11 SNPs) and OP508345_1_LSDV_China/Xinjiang/Cattle/2019 (23 SNPs) and HongKong MW732649_1_LSDV/HongKong/2020 (76 SNPs). Other notable LSDVs in recombinant_3-like cluster were OM793602_1_LSDV_Russia_Tomsk_2020 and OM793603_1_LSDV_Russia_Khabarovsk_2020 from Russia with 11 and 24 SNPs respectively (Fig. 4C).

The recombination analysis of the LSDV genomes on RDP4 revealed 11 potential recombination events in the Indonesia LSDV genome, with LSDV_Neethling_Indoevaccine_LW_1959 (AF409138.1) and LSDV_NI-2490 (AF325528.1) as the parental donors. Similar recombination events are also present in other LSDV isolates from China, Thailand, Hong Kong, Vietnam, Taiwan, and Tomsk, Russia (Table 1). Some of the ORFs affected by the recombination events include the G-protein coupled receptor gene (LSDV011), the DNA polymerase gene (LSDV039), the DNA ligase-like protein gene (LSDV133), and the variola virus B22R-like protein gene (LSDV134). These four ORFs are among the nine proteins annotated as only 99.5-99.9% homologous to both LSDV_NI-2490 and LSDV_Neethling_vaccine_LW_1959 reference genomes (Additional file 1: Table s3). The most notable ORF was LSDV087, whose sequence analysis showed a nucleotide insertion at position 599 in all Neethling vaccine strains which caused a frameshift and truncated the ORF to 603nt instead of 763nts. No recombination was observed within this ORF, and all known LSDV recombinants were 100% identical to the field isolates in this region of the genome. Further, in addition to the 8 SNPs observed between the LSDV NI-like field isolates and Neethling-like field isolates, the nucleotide alignment of ORF087 showed 14 SNPs between the LSDV NI-like field isolates and Neethling derived vaccines; and 7 SNPs between Neethling-like field isolates and Neethling derived vaccines (Additional file 2: Figure s1).

**Table 1:** Predicted potential recombination events using different detection methods available in the RDP4 program

	Breakpoint positions in the alignment					*P*-Values for the detection methods in RDP4							
Event Number	Begin	End	Recombinant Sequence(s)	Minor Parental Sequence(s)	Major Parental Sequence(s)	ORF	RDP	GENECONV	Bootscan	Maxchi	Chimera	SiSscan	3Seq
1	134,852	139,255	LSDV_Indonesia_2022_S1/S4	AF325528.1_LSDV_NI-2490	AF409138.1_LSDV_Neethling_vac_1959	*LSDV143-LSDV145*	*5.73E-65*	*5.84E-64*	*1.98E-47*	*6.88E-26*	*7.81E-22*	*3.41E-27*	*1.51E-13*
2	1716	4457	LSDV_Indonesia_2022_S1/S4	AF325528.1_LSDV_NI-2490	AF409138.1_LSDV_Neethling_vac_1959	*LSDV003-LSDV007*	*1.58E-20*	*4.97E-21*	*4.78E-14*	*3.69E-11*	*1.13E-11*	*1.54E-06*	*NS*
3	120,400	122,490*	LSDV_Indonesia_2022_S1/S4	AF325528.1_LSDV_NI-2490	AF409138.1_LSDV_Neethling_vac_1959	*LSDV133-LSDV134*	*1.85E-14*	*1.72E-12*	*4.92E-07*	*5.17E-10*	*5.16E-10*	*7.12E-05*	*5.61E-09*
4	95,204	99,293	LSDV_Indonesia_2022_S1/S4	AF325528.1_LSDV_NI-2490	AF409138.1_LSDV_Neethling_vac_1959	*LSDV101-LSDV104*	*2.50E-14*	*1.34E-14*	*1.90E-07*	*2.75E-07*	*2.73E-07*	*NS*	*2.04E-10*
5	139,877*	144,009	LSDV_Indonesia_2022_S1/S4	AF325528.1_LSDV_NI-2490	AF409138.1_LSDV_Neethling_vac_1959	*LSDV146-LSDV149*	*1.80E-13*	*2.34E-09*	*6.64E-04*	*4.45E-12*	*4.42E-12*	*NS*	*8.06E-11*
6	19,712	23,339	LSDV_Indonesia_2022_S1/S4	AF325528.1_LSDV_NI-2490	AF409138.1_LSDV_Neethling_vac_1959	*LSDV027-LSDV031*	*5.57E-11*	*3.83E-11*	*1.25E-10*	*2.45E-03*	*2.44E-03*	*2.75E-02*	*1.15E-07*
7	91,356	92,836	LSDV_Indonesia_2022_S1/S4	AF325528.1_LSDV_NI-2490	AF409138.1_LSDV_Neethling_vac_1959	*LSDV098*	*6.75E-11*	*1.04E-09*	*2.17E-09*	*1.86E-05*	*9.93E-06*	*NS*	*NS*
8	85,665	89,204	LSDV_Indonesia_2022_S1/S4	AF325528.1_LSDV_NI-2490	AF409138.1_LSDV_Neethling_vac_1959	*LSDV091-LSDV094*	*7.73E-12*	*1.18E-08*	*2.68E-03*	*1.25E-05*	*1.05E-05*	*NS*	*4.55E-07*
9	7070	9986	LSDV_Indonesia_2022_S1/S4	AF325528.1_LSDV_NI-2490	AF409138.1_LSDV_Neethling_vac_1959	*LSDV011-LSDV014*	*9.63E-12*	*3.19E-09*	*1.10E-06*	*5.44E-07*	*9.18E-07*	*NS*	*2.84E-07*
10	48,555	53,550	LSDV_Indonesia_2022_S1/S4	AF325528.1_LSDV_NI-2490	AF409138.1_LSDV_Neethling_vac_1959	*LSDV054-LSDV060*	*1.19E-13*	*4.16E-07*	*5.78E-05*	*2.49E-04*	*1.19E-06*	*NS*	*7.56E-11*
11	33,932	35,060	LSDV_Indonesia_2022_S1/S4	AF325528.1_LSDV_NI-2490	AF409138.1_LSDV_Neethling_vac_1959	*LSDV039*	*4.75E-09*	*3.39E-08*	*8.10E-06*	*NS*	*1.10E-02*	*NS*	*6.24E-04*

* = The actual breakpoint position is undetermined (it was most likely overprinted by a subsequent recombination event)

NS = Not significant

**Fig. 5:**
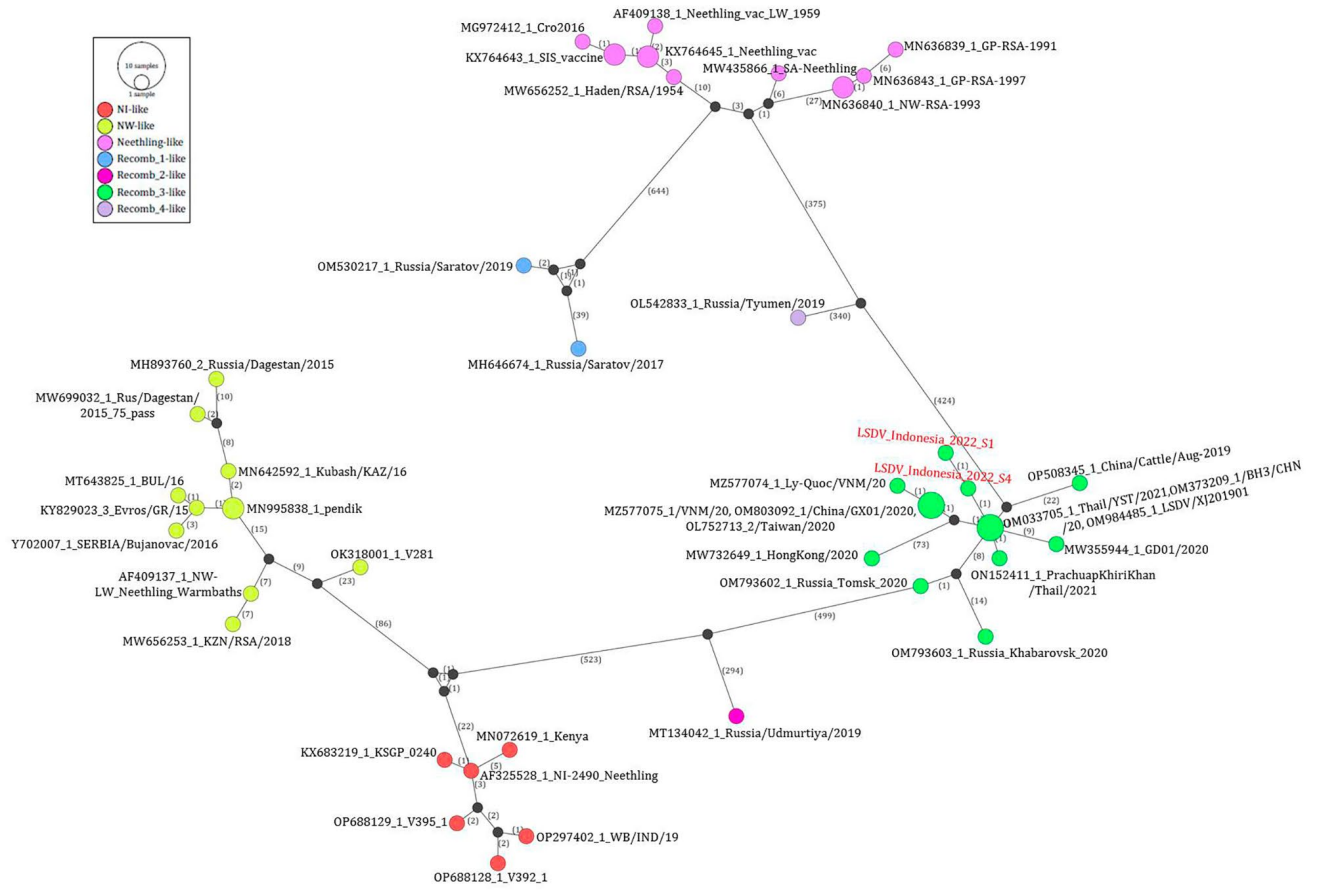
Median-joining network inferred from LSDV whole genome sequences using the PopART program. The network shows the Indonesia LSDVs (in red) in cluster Recomb_3-like, between the NI-like and Neethling-like LSDV clusters. The number of mutations between each genome is labeled

The phylogenetic analysis of sequence fragments located on the 5’ and 3’ ends of breakpoint events supported the recombination events. For example, analysis of the sequences located on the 5’ end of the breakpoint event 10, clustered the Indonesian LSDVs with LSDV_NI-2490 (Cluster II), while the sequences on the 3’ end of the breakpoint clustered them with LSDV_Neethling_vaccine_LW_1959 (Cluster I) (Additional file 3: Figure s2). The recombination of Indonesian LSDVs was also confirmed using SimPlot, followed by phylogenetic analysis of the sequences located on the 5’ and 3’ ends of the breakpoints (Additional file 4: Figure s3). Similarly, the phylogenetic network analysis of the aligned LSDV genomes using PopART software indicated that the Indonesia LSDVs are likely recombinants of NI-like and Neethling-like LSDVs placed on the opposite ends of the network. The network confirmed that the LSDVs from Indonesia cluster together with other isolates from East and Southeast Asia (recomb_3-like), which have been demonstrated as LSDV recombinants and are different from the known LSDV recombinants from Russia (recomb_1-like, recomb_2-like, and recomb_4-like) (Fig. 5).

## Discussion

This study has presented typical clinical signs of LSD including skin nodules, nasal discharge, and high fever in suspected cattle cases in 2022 outbreak in Indragiri Hulu district, Indonesia, with morbidity and mortality rates of 28.04% and 1.87%, respectively. It has been reported that the severity of LSD varies based on the immune status of the animal, the breed, the age, and the virus strain, leading to an average mortality rate of < 10% and variable morbidity rate from 5 to 45% [2, 4]. However, the morbidity and mortality rates recorded in the LSD outbreak in Indonesia were consistent with other LSD outbreaks in Asian countries [28, 37, 38].

The initial molecular characterization of the Indonesian LSDV isolates based on targeted CaPV-marker genes indicated possible virus recombination [18–21]. First, the nucleotide sequence analysis of the four targeted genes showed that the Indonesian LSDV isolates were 100% identical to the recently demonstrated LSDV recombinants from East and Southeast Asia: China, Thailand, Taiwan, Vietnam, and HongKong [23, 32–35]. Second, on one hand, the complete RPO30 gene and the partial EEV gene sequence analysis showed that Indonesian LSDV sequences have 99.67% and 100% sequence similarity, respectively, to the LSDV Neethling-derived vaccines. On the other hand, the GPCR gene sequence similarity was highest (99.13%) to the common LSDV field strains, while the B22R sequence was 99.40% similar to the Neethling vaccine strain and 99.28% to the NI-2490 LSDV strain.

Further analysis of the Indonesian LSDV whole genome sequences confirmed its closest homology to the recombinant LSDVs from East and Southeast Asia and that it was 99.56% similar to Neethling_LSDV_vaccine and 99.25% to the LSDV_NI-2490 strain. Possible recombination of the Indonesian LSDV genomes was assessed and verified using different tools (RDP4, SimPlot, DAPCA, and PopART network) that have previously been applied in recombination analysis [39, 40]. The PopART network and the PCA scatter plot showed seven clusters of LSDVs and grouped Indonesia isolates with other reported East and Southeast Asia recombinant LSDVs [41]. The RDP4 and SimPlot indicated that Indonesia LSDVs are most likely recombinants of Neethling_LSDV_vaccine and LSDV_NI-2490 isolates with 11 recombination events. Notably, the recombinant events detected in this study have been reported in LSDV genomes from Thailand and China [32, 34]. Although the parental donors for all the reported LSDV recombinants are Neethling vaccine and Kenyan NI-2490-like strains, the Indonesia LSDV recombination was similar to other recombinants from East and Southeast Asia but different from those found in Russia that have 27 (LSDV_ Russia/Saratov/2017), 24 (LSDV_Udmurtiya_Russia_2019) and 25 (Tyumen_2019, OL542833_1) recombination events [42–44]. The difference in recombination patterns of LSDVs was demonstrated in this study by the SNP heatmap and the PopART network analysis that showed the phylogenetic relatedness of the recombinants to their parental donors.

In addition to the previously reported genes employed in combination in this study (GPCR, RPO30, EEV, and B22R), the LSDV whole genome analysis suggested that LSDV087 could be an additional DIVA target through PCR and sequencing, that can complement the existing CaPV RT-PCR DIVA methods, due to the unique signatures or SNPs found at DNA level in LSDV Neethling derived vaccines compared to the field strains including the LSDV recombinants [18–21]. In addition to the intergenic recombination (exchange of full-length gene sequence) observed in Indonesian LSDV genomes and other reported recombinant LSDVs, intragenic recombination (exchange of gene sequence fragments) was also detected in nine genes [45]. Recombination in viruses is common in the field following a co-infection of viruses in a single cell of a host which is influenced by the selection pressure of a virus population and contributes to virus evolution through genetic shift [45, 46]. The intragenic recombinations like those detected in the Indonesia LSDV isolates are related to the regulation of gene expression, hence altering the pathogenic properties of the virus [45, 46]. Virus recombination in the field is likely following vaccination with live attenuated vaccine in a population where wild-type virus is already circulating or contamination in cell culture during vaccine production [47]. No LSDV vaccination program was reported in Indonesia before the LSD outbreak; therefore, it is possible that the virus was introduced into the country from the neighboring countries. This is likely considering that the Indonesian LSDV isolate is nearly identical to the isolates circulating in the neighboring countries in East and Southeast Asia, suggesting a common origin of the virus in the region. A recent study has shown that the emergence of LSDV recombinants in Asia is most likely a result of a spillover from animals vaccinated with the Lumpivax vaccine that appears to be a mixture of Neethling-like LSDV vaccine strain, KSGP-0240 vaccine strain and several LSDV recombinants that are nearly identical to those circulating in Asia [41]. For instance, the initial outbreak of the recombinant_3-like LSDVs was reported in Qapqal Xibe county, Xinjiang province in Northwest China, which is located ∼ 20 km from the border of Kazakhstan, where a mass vaccination campaign with Lumpivax vaccine was launched [48]. Thus, it is likely that in addition to the poor-quality vaccine spillover, cattle movement, trade and insect vectors may have contributed to the spread of the LSDV recombinants into the neighboring countries in the region. Moreover, It would be interesting to further investigate whether the changes in proteins for viral virulence and host range, such as G-protein coupled receptor (LSDV011), IFN-alpha beta receptor glycoprotein (LSDV135), IL-10 (LSDV005) and IL-1 receptor (LSDV006), that were found in LSDV recombinants circulating in Indonesia and other Southeast Asian countries cause higher infectivity and virulence, thereby contributing to the virus’s rapid spread as suggested in earlier studies with LSDV recombinants in Russia [49, 50].

## Conclusion

This study has reiterated the importance of applying several analytical tools for accurate LSDV whole genome analysis, especially with datasets including recombinants. Based on these tools, the study has shown that the Indonesia LSDV isolate is a recombinant of Neethling_vaccine_LW_1959 and LSDV_NI-2490 strains and is closely related to the previously reported LSDV recombinants circulating in East and Southeast Asia, suggesting a common origin/source of the LSDV isolate causing the outbreak and spreading in the region. Most importantly, the analysis in this study has identified a unique ORF, LSDV087, as a potential DIVA target for LSDVs through sequencing. Therefore, this study demonstrates the significance of accurate molecular characterization of LSDV for disease surveillance and understanding of its origin, as well as vaccine quality control before animal vaccination.

## Materials and methods

### Sample collection and processing

Six skin nodules and six nasal swab samples were collected from suspected cattle cases in Indragiri Hulu district in 2022 and sent to the RCVS for further investigation. DNA was extracted using the Qiagen AllPrep DNA/RNA extraction kit and eluted in 50 μl elution buffer.

### PCR and differential diagnosis

A real-time PCR (RT-PCR), previously described by Bowden et al. [6], was used to detect the CaPV genome in all twelve collected samples. The PCR reactions were prepared in a 20 μl reaction volume containing 18 μl of the PCR master mix with primers, a probe, and two μl of template DNA. The positive samples were characterized further using the high-resolution melting (HRM) assay capable of differentiating CaPVs based on the melting temperature (Tm) of the PCR amplicon [51].

### Amplification and sequencing of selected CaPV genes

Four selected CaPV-marker genes (RPO30, GPCR, EEV, and B22R) that can differentiate LSDV vaccine strains from field isolates were amplified and sequenced for the CaPV-positive samples following the previously described PCR conditions [52]. The PCR products were analyzed on a 2% agarose gel in 1X TAE buffer and visualized on a Gel Documentation System (Bio-Rad). The PCR products were purified and sent for Sanger sequencing from both directions using forward and reverse primers at LGC Genomics (Germany).

### Whole genome sequencing of LSDV using Ion S5 technology

Approximately 100 ng of DNA was enzymatically fragmented into 200 bp lengths using Ion shear Plus reagents. The fragmented DNA was ligated to adapters and barcodes using the Ion Xpress™ Plus Fragment Library Kit and the Ion Xpress barcode adapters (Thermo Fisher Scientific, USA) following the manufacturer’s instructions. Size selection was performed using Pippin Prep (Sage Science, Inc., USA). The libraries were further amplified for eight cycles using Platinum™ PCR SuperMix high fidelity and library amplification primer mix provided with the Ion Plus Fragment Library Kit. The amplified barcoded libraries at a concentration of 100 pM were pooled in equal volumes and loaded onto the Ion Chef™ Instrument (Thermo Fisher Scientific, USA) for automated template preparation and chip loading using Ion 540™ Kit-Chef (Thermo Fisher Scientific, USA). Using the Ion Chef™ Instrument, the pooled libraries were clonally amplified on the Ion spheres (ISPs) by emulsion PCR, followed by automated loading template-enriched ISPs onto an Ion 540 chip. Sequencing was performed on an Ion S5™ Next generation sequencing system (Thermo Fisher Scientific, USA) with 500 flows to generate 200 bp reads.

### Sequence and phylogenetic analysis of CaPV-marker genes

The obtained sequences were assembled using Vector NTI software (Invitrogen) v11.5. For each targeted gene, the sequences were analyzed with those of other LSDVs, SPPVs, and GTPVs retrieved from GenBank. Multiple sequence alignments were performed on MEGA X using the Muscle algorithm and the codon option. Neighbor-joining trees for the complete RPO30 and GPCR genes were also built on MEGA X, and the evolutionary distances were computed using the Maximum Composite Likelihood method with 1000 bootstrap replicates [53]. The phylogenetic trees with the associated metadata were visualized on the Interactive Tree of Life (ITOL) tool [54]. The multiple sequence alignments, analysis, and visualization of the partial EEV glycoprotein and B22R genes were conducted using BioEdit v7.2.5.

### Whole genome reconstruction and annotation of the Indonesian LSDV isolate

The run was pre-processed using the torrent suite software to remove adapters. Then, the raw reads were quality-filtered using fastq-mcf v1.04.676 (ea-utils), and their quality was assessed with FastQC (v. 011.5). A Phred score cut-off of 20 was used, and the reads with lengths between 50 and 250 bp were selected. Denovo assemblies were performed on a subset of reads using SPAdes (v3.11.1), Unicycler (v0.5.0), and Megahit (v1.2.9). Reference-guided assembly was performed using bowtie2 (2.3.4.1). After mapping the cleaned raw reads against the reference sequence, the Mpileup files were generated using SAMtools (v1.11). Variant calling was performed using the consensus caller method of BCFtools (v1.9). The consensus sequences were produced with vcfutils.pl (VCFtools v0.1.16) and seqtk (v1.3.106) using a Phred score of 20 and compared to the de novo assemblies after alignment with Mafft (v7.453). The assemblies were also visualized using IGV, and mapping statistics were visualized with Qualimap (v.2.2.1).

Given that the Indonesia LSDVs were grouped in LSDV Cluster I or II based on different LSDV-marker genes analysis, the open reading frames (ORFs) of the assembled Indonesia LSDV genomes were predicted with GATU using reference genomes from LSDV Cluster I; LSDV_Neethling_vaccine_LW_1959 (AF409138.1) and Cluster II; LSDV_NI-2490 (AF325528.1) [55]. The whole genome sequences of Indonesia LSDV isolates, LSDV_Indonesia_2022_S1, and LSDV_Indonesia_2022_S4, were deposited in the GenBank database under the accession numbers OR232413 and OR232414, respectively.

### LSDV whole genome SNP and recombination analysis

The assembled Indonesian LSDV genomes were aligned with 46 complete LSDV genome sequences retrieved from GenBank using MAFFT. The SNPs from the aligned LSDV dataset relative to the LSDV_NI-2490 (AF325528.1) genome were analyzed using the adegenet package in R. First, the alignment file was converted into a genlight object using the function fasta2genlight and the SNPs were extracted [56]. The SNPs distribution and density of the LSDV genotypes were visualized by heatmap. The discriminant analysis of Principal Component Analysis (DAPCA) of the extracted SNPs was also performed to identify the different clusters of the LSDV genotypes, which were plotted as a PCA scatterplot and neighbor-joining tree. Further SNP-based analysis was performed on all LSDV genomes clustering together with the Indonesia LSDV isolates. Three different tools were applied to assess evidence of recombination in the Indonesia LSDV isolate. The potential recombination events of the Indonesia LSDV isolates were first evaluated using the RDP4 software, which includes seven methods (RDP, GENECONV, BootScan, MaxChi, Chimera, SiScan, and 3Seq) [57]. The recombination was then analyzed using the SimPlot 3.5.1 software with a window size of 1000 bp and a step of 20 bp. Given that recombinant genome sequences are not suitable for whole genome phylogenetic analysis [25, 27], several phylogenetic trees were constructed based on the fragment sequences before and after the breakpoints predicted by RDP4 and SimPlot software on MEGA X, using the neighbor-joining method with 1000 bootstraps and visualized on ITOL. Lastly, the recombination was explored by conducting a phylogenetic network analysis of the aligned LSDV genomes on the PopART program, using the median-joining method with epsilon set to zero [40].

### Data availability

The DNA sequences generated and used in the analysis for this study are available in the Gen-Bank under the accession numbers OR246967 to OR246986 for gene fragments and OR232413 and OR232414 for the whole genomes.

### Electronic supplementary material

Below is the link to the electronic supplementary material.

Supplementary Material 1

Supplementary Material 2

Supplementary Material 3

Supplementary Material 4

## Abbreviations

BCF: Binary variant call format
IGV: Integrative genomics viewer
LSDV: Lumpy skin disease virus
PCA: Principal Component Analysis
SAM: Sequence Alignment/Map
SNP: Single nucleotide polymorphism
VCF: Variant call format
