# Supplementary material for: Molecular characterization of recombinant LSDV isolates from 2022 outbreak in Indonesia through phylogenetic networks and whole-genome SNP-based analysis
Source: BMC Genomics. 2024 Mar 4;25:240. doi: 10.1186/s12864-024-10169-6 (PMC10913250; doi:10.1186/s12864-024-10169-6)
Supplement: Supplementary file 2 — Supplementary Material 2 [file 12864_2024_10169_MOESM2_ESM.docx]

a). Nucleotide alignment


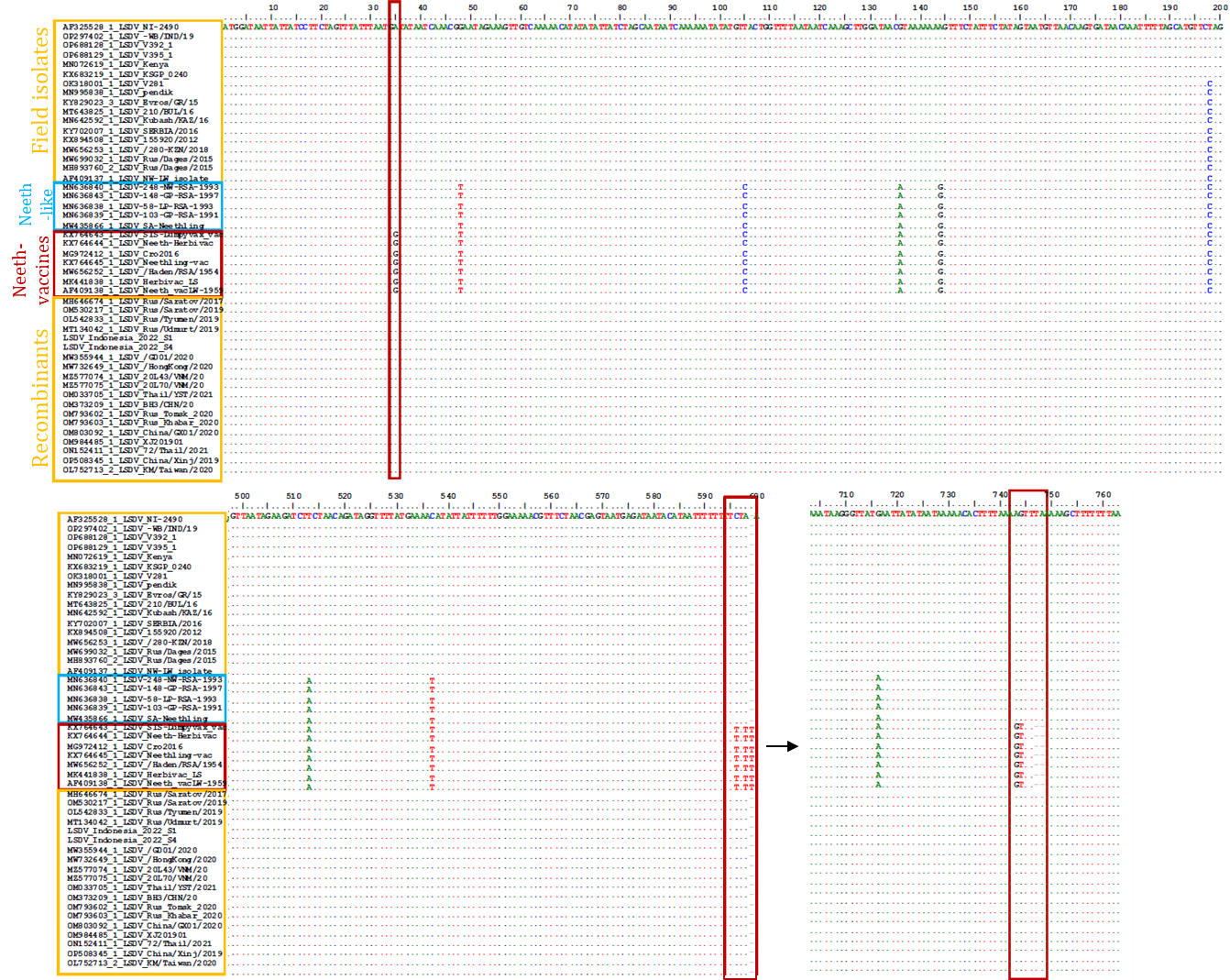


b). Amino acid alignment


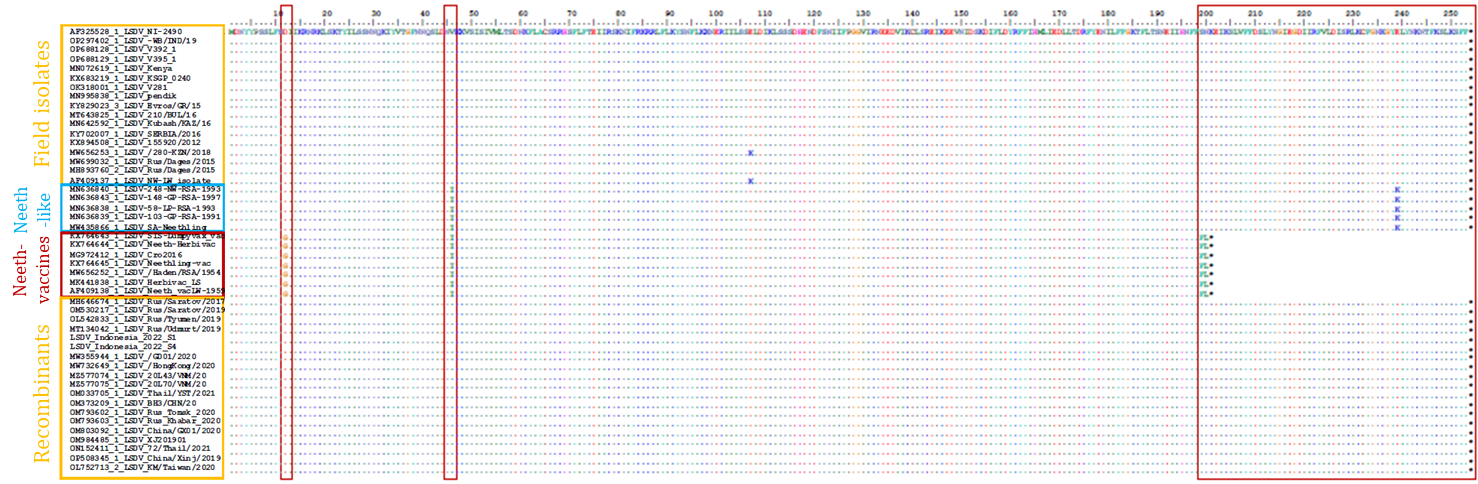


**Additional file 2: Figure s1.** Multiple sequence alignment of the LSDV087 (mRNA decapping enzyme (Cop-D10R)), a potential DIVA target for LSDVs. (a) nucleotide alignment showing the InDels and SNPs in the LSDV087 of the Neethling vaccine strains and (b) amino acid alignment showing the truncated LSDV087 protein of the Neethling vaccines. The dots indicate the identical nucleotides/amino acids in the alignments.
