# Supplementary figures and images for: Molecular characterization of recombinant LSDV isolates from 2022 outbreak in Indonesia through phylogenetic networks and whole-genome SNP-based analysis
Source: BMC Genomics. 2024 Mar 4;25:240. doi: 10.1186/s12864-024-10169-6 (PMC10913250; doi:10.1186/s12864-024-10169-6)

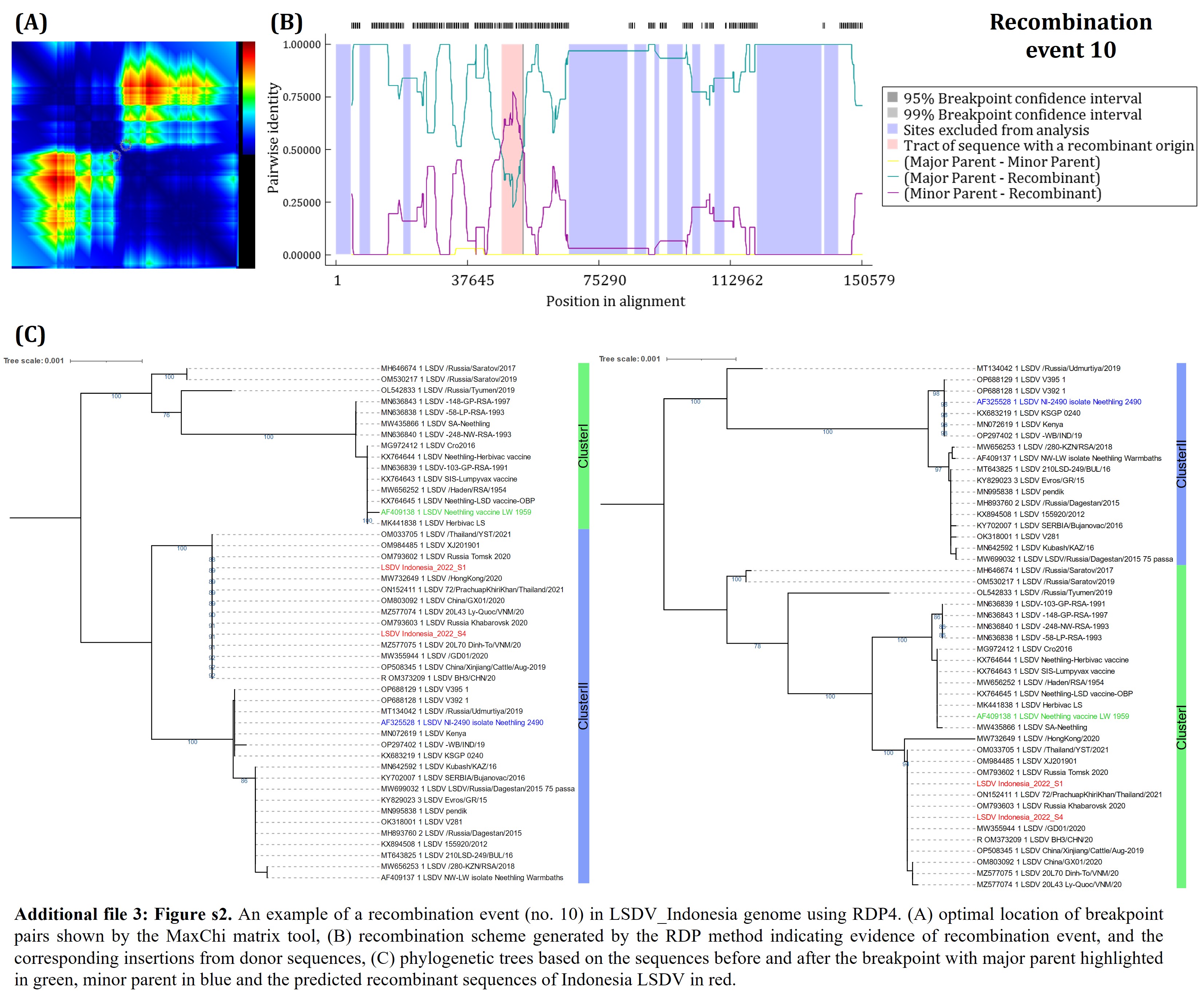

Supplement: Supplementary file 3 — Supplementary Material 3 [file 12864_2024_10169_MOESM3_ESM.jpg]

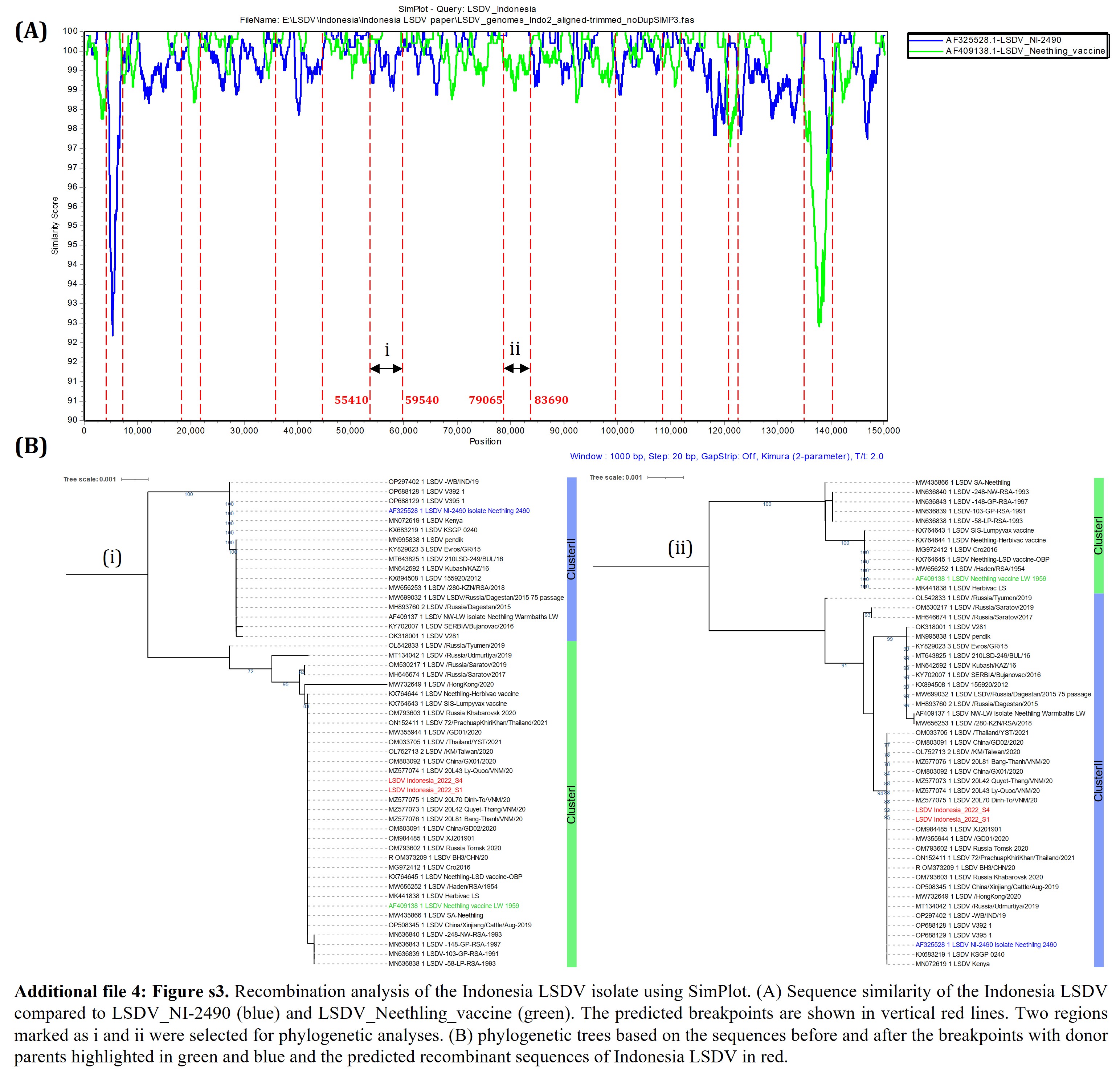

Supplement: Supplementary file 4 — Supplementary Material 4 [file 12864_2024_10169_MOESM4_ESM.jpg]
